# RRBP1 depletion of bone metastatic cancer cells contributes to enhanced expression of the osteoblastic phenotype

**DOI:** 10.3389/fonc.2022.1005152

**Published:** 2022-12-09

**Authors:** Rui Chen, Yue Wang, Yang Xu, Yaohui He, Qing Li, Chun Xia, Bing Zhang

**Affiliations:** ^1^ Cancer Research Center, School of Medicine, Xiamen University, Xiamen, Fujian, China; ^2^ Bone & Joint Research Institute, Zhongshan Hospital, School of Medicine, Xiamen University, Xiamen, Fujian, China; ^3^ School of Pharmaceutical Sciences, Xiamen University, Xiamen, Fujian, China

**Keywords:** RRBP1, conditioned mediums (CMs), bone metastatic cancer cells, the osteoblastic phenotype expression, MC3T3-E1 cells, endoplasmic reticulum (ER) stress

## Abstract

Bone metastatic cancer-secreted extracellular factors are capable of modifying the bone microenvironment through interacting with bone cells, including osteoblasts. Reticulum ribosome-binding protein 1 (RRBP1) is substantially expressed in certain bone metastatic cancer cells. This study was undertaken to determine whether RRBP1 from bone metastatic cancer cells affects the osteoblastic phenotype expression. Breast and prostate cancer cells, MDA-MB-231 and PC3, were cultured, respectively, followed by collecting conditioned mediums (CMs) and identifying the abundance of RRBP1 in CMs using LC-MS/MS. MC3T3-E1 cells were cultured with a mixed medium (including CMs from sh*RRBP1*-transduced two-type cancer cells) with or without endoplasmic reticulum (ER) stress inhibitor 4-PBA, followed by measuring the levels of osteoblastic phenotype expression and biomarkers of ER stress using western blotting, qPCR, and ARS staining, respectively. Similar experiments were performed in sh*Rrbp1*-transduced MC3T3-E1 cells cultured with a mixed medium (including CMs from the two-type cancer cells). Bone formation parameters were measured in the tibia of nude mice injected with sh*RRBP1*-transduced two-type cancer cells using micro-CT analysis. These results showed that RRBP1 is the sole shared high-abundance protein in CMs from the two-type cancer cells, involving osteoblast differentiation. CMs from sh*RRBP1*-transduced two-type cells boosted the osteoblastic phenotype expression partially through increasing ER stress. CMs from the two-type cancer cells partially offset the similar alterations induced by sh*Rrbp1* in MC3T3-E1 cells. Injection with sh*RRBP1*-transduced two-type cells ameliorated the bone lesions in nude mice. Therefore, RRBP1 depletion of bone metastatic cancer enhanced the osteoblastic phenotype expression, suggesting a role of RRBP1 in the bone microenvironment.

## Introduction

Bone metastasis is a key element of morbidity and mortality in most patients with advanced cancers, resulting in osteolytic (excess bone resorption)-type lesions, osteoblastic (excess bone formation)-type, and mixed lesions (1). The imbalance between bone resorption and bone formation in the bone microenvironment contributes to bone metastatic lesions (1). Numerous studies have demonstrated that cancer-secreted extracellular factors are capable of modifying the bone microenvironment through interacting with bone cells, including osteoblasts (responsible for bone formation) and osteoclasts (responsible for bone resorption). For example, breast cancer cells metastasize to bone tissue and produce many factors to mediate bone destruction by stimulating osteoclasts (1, 2). Prostate cancer cells supply several osteoblast-stimulating factors to osteoblasts or osteoblast precursor cells to promote bone formation in the bone microenvironment (3–5). Although the role of osteoclasts in the bone microenvironment seems to be more important than that of osteoblasts, increasing evidence report that osteoblasts might play equal, even more, important roles than osteoclasts during bone metastasis of cancer cells. The addition of bisphosphonates, which is aimed at impairing the activity of bone-resorbing osteoclasts, does not extend disease-free survival for patients with osteolytic bone metastatic breast cancer (6). Based on their studies, Dr. Andrea M. Mastro and his colleagues put forward the idea that osteoblasts are more than intermediaries between tumor cancer cells and osteoclasts, as osteoblasts are co-opted into creating a microenvironment that exacerbates bone loss and are prevented from producing matrix proteins for mineralization (7–9). Therefore, the influence of cancer-secreted extracellular factors on osteoblasts is worthy of more attention.

Reticulum ribosome-binding protein 1 (RRBP1), an endoplasmic reticulum (ER) membrane protein, is essential for ribosome binding and the translocation of nascent proteins across the membrane of rough ER (10–12). In addition, RRBP1 is detected in the nucleus and cytoplasm (13), which is involved with the localization of a subset of mRNAs to ER in a ribosome-independent manner to assist in the translation of mRNAs and secretion of various protein products (14). As a potential oncogene, increased RRBP1 expression has been linked to certain bone metastatic cancer cells, including lung, breast, and prostate cancers (15–17). Recently, Zheng et al. reported that RRBP1, together with COL6A1, VCAN, and CREB3L, is annotated as osteoblast differentiation in periodontal ligament stem cells (18). However, whether RRBP1 of bone metastatic cancer cells is responsible for modifying the phenotype expression of osteoblasts in the bone microenvironment remains unknown.

In this study, we identified the abundance of RRBP1 protein in the conditioned mediums (CMs) from human breast cancer cell line MDA-MB-231 and prostate cancer cell line PC3, respectively. We also looked into the effect of CMs from the two-type cancer cells transduced with the shRNA/*RRBP1* vector (sh*RRBP1*) on the osteoblastic phenotype expression in MC3T3-E1 preosteoblastic cells, the effect of injection with the two-type cancer cells transduced with sh*RRBP1* into the tibia medullary cavity of nude mice on bone tissue, and the mechanism of RRBP1 responsible for modifying the osteoblastic phenotype expression associated with ER stress. Our data suggest that depletion of RRBP1 in bone metastatic cancer cells could boost the osteoblastic phenotype expression, partially through the enhancement of ER stress.

## Materials and methods

### Cell culture

Bone metastatic human breast cancer MDA-MB-231 and prostate cancer PC3, and embryonic kidney HEK293T cell lines were obtained from the Shanghai Institute of Cell Biology, Chinese Academy of Sciences (Shanghai, China). MDA-MB-231 and HEK293T cells were maintained in DMEM with high glucose (Hyclone, Logan, UT, USA), and PC3 cells were maintained in F12 (Hyclone, Logan, UT, USA). The mouse preosteoblastic cell line, MC3T3-E1, purchased from ATCC (Manassas, VA, USA) was cultured in α-MEM (Hyclone, Logan, UT, USA). The mediums were supplemented with 10% fetal bovine serum,100 U/mL penicillin, and 100 μg/mL streptomycin, and the cells were cultured at 37°C in a water-saturated atmosphere of 5% CO_2_.

### Preparation of cancer cell-CMs

MDA-MB-231 or PC3 cells were seeded at a density of 1×10^7^ cells/dish of a 150 mm petri dish in DMEM with high glucose or F12 for 24 h, respectively. After the seeded cells reached 80% confluency, the medium was filtered by 0.44 μm filter and collected as cancer cell-CM from MDA-MB-231 or PC3 cells, respectively. According to subsequent experimental designs, MC3T3-E1 were cultured in a 1:1 mixture of double mineralized medium (α-MEM, containing 10 nM dexamethasone, 2 mM β-GP, and 50 μg/mL vitamin C) and CM from MDA-MB-231 cells (CM-231) or CM from PC3 cells (CM-PC3) as described previously (9, 19).

### Mass spectrometry sample preparation

MDA-MB-231 or PC3 cells were seeded at a density of 1 ×10^7^ cells/dish of a 150 mm petri dish in a serum-free medium for 48 h, respectively, which reached 80% confluency. 15 mL CM-231 or CM-PC3 was collected and centrifuged at 3000 x g for 5 min to remove the dead cells and cell debris, respectively. The supernatants were concentrated to 1 mL in the filter unit (Amicon Ultra-4, Millipore, MA, USA) and prepared for LC-MS/MS analysis as described in previous studies (20, 21). Briefly, the supernatants were reduced with 8 M urea (UA), 10 mM DTT, and 50 mM iodoacetamide (IAA), digested with ice-cold 0.01 μg/μL trypsin in 20 mM ammonium bicarbonate buffer (ABC), acidified with 50 μL 50% acetonitrile (ACN)/1% formic acid (FA), and desalted with 0.1% FA and C18. The samples were pooled, dried in a Speedvac (Eppendorf, German), and resuspended with 0.1% FA. The concentration of samples was detected before liquid chromatography-tandem mass spectrometry (LC-MS/MS) analysis.

### LC-MS/MS analysis

Orbitrap Fusion Lumos equipped a nanoelectrospray source combined with a nanoscale EASY-nLC 1200 UHPLC system (Thermo Fisher Scientific) was used to perform all MS experiments, as described previously (20, 21). Briefly, CM from MDA-MB-231 cells (CM-231) or CM from PC3 cells (CM-PC3), which was prepared as described above, was separated by a nanoscale RP-HPLC column (75 µm × 25 cm) packed with 2 µm C18 beads. The mobile phase A and B were prepared using 0.1% FA and 80% MS-grade ACN dissolved in double-distilled water. The separation flow rate was set at 350 nL/min and the gradient applied to ranging from 9% to 29% of mobile phase B over 95 min followed by a linear increase to 44% of mobile phase B. A data-dependent manner alternating between full-scan MS and MS2 scans was carried out to acquire raw data. The spray voltage was set at 2.2 kV. The temperature of the ion transfer tube was 300°C. Full scans ranging from 350 to 1800 m/z with 120,000 resolutions were recorded in the Orbitrap analyzer. The Target value for AGC was set to 4 × 10^5^, and the maximal injection time was set to 50 ms. Selected ions were sequentially fragmented in a 3 seconds cycle by HCD with 30% normalized collision energy, specified isolated windows 1.6 m/z, 15,000 resolutions. Unassigned ions or those with a charge of 2+ and >7+ were rejected for MS/MS. AGC of 5 × 10^4^ and 40 ms maximal injection time were used. Dynamic exclusion was set to 40 s. Raw data were processed and annotated using Proteome Discoverer (PD, version 2.2) and reviewed by the SwissProt human proteome database (20259 entries). Only peptides with at least six amino acids in length were considered. The peptide and protein identifications were filtered by PD to control the false discovery rate (FDR) <1%. At least one unique peptide was required for protein identification.

### Plasmid construction and transfection

Briefly, PLKO.1 vector was digested with EcoR1(New England BioLabs, Beijing, China) and Age1(New England BioLabs, Beijing, China). The reaction product was recovered with the HiPure Gel Micro Kit (Magen, Guangzhou, China) after Agron gel electrophoresis. The short hairpin RNA sequences for RRBP1 were shown in **Table 1**. The short hairpin RNA targeting the *RRBP1* vector (sh*RRBP1*) was then ligated with T_4_ DNA ligase. A stable cell line was established under the pressure of puromycin (5 μg/mL, APExBIO, Huston, USA) after lentiviral transfection with sh*RRBP1* or sh*RRBP1*-2 (22). Before further investigations, the level of RRBP1 was determined by qPCR and western blotting assays. The luciferase reporter gene was expressed by transfecting the pLenti X2 Hygro/pTER shLUC (w607-1) vector into the animal research cells, and stable cell lines were established under the pressure of hygromycin B (0.2 mg/mL, Yeasen, Shanghai, China) and puromycin (5 μg/mL, APExBIO, Huston, USA) (23).

**Table 1:** The short hairpin RNA sequences for RRBP1.

Gene name	Primer sequences
H-sh*RRBP1*-1	5’-ccgg GTGAAGCATCTCGAAGAGATT ttcaagaga AATCTCTTCGAGATGCTTCAC tttttg-3’
H-sh*RRBP1*-2	5'-ccggGACACCAACAAGATTGAGGAAttcaagaga TTCCTCAATCTTGTTGGTGTC tttttg-3’
M-sh*Rrbp1*	5’-ccggGCAGTCAGTTCTATTGTGAATttcaagaga ATTCACAATAGAACTGACTGC tttttg-3’

### Quantitative real-time PCR assay

Total RNA was extracted by using an RNA extraction kit (Bioflux, Beijing, China) and then subjected to reverse transcription by using Primescript RT Master Mix Kit (Takara, Dalian, China) to synthesize cDNA. Real-time PCR was then performed using a Light Cycler (Roche) with a SYBR Premix Ex Taq II Kit (Takara, Dalian, China). Results were analyzed as described in the previous study (24). The primers used for qPCR was described in **Table 2**.

**Table 2:** The primers used for quantitative PCR.

Gene name	Primer sequences
M-*Rrbp*1	Forward 5’-GGAAGATACCTGAACATGACCTG-3’Reverse5’- CCACCATAGGCACCTCCTT -3’
M-*ALP*	Forward 5’-GTTGCCAAGCTGGGAAGAACAC-3’Reverse5’-CCCACCCCGCTATTCCAAAC-3’
M-*BGLAP*	Forward 5’-GAACAGACTCCGGCGCTA-3’Reverse5’-AGGGAGGATCAAGTCCCG-3’
M-*TNFRSF11B*	Forward 5’-ACCCAGAAACTGGTCATCAGC-3’Reverse5’-CTGCAATACACACACTCATCACT-3’
M-*TNFSF11*	Forward 5’-CTGATGAAAGGAGGGAGCACG-3’Reverse5’-AGCAGGGAAGGGTTGGACAC-3’
M-*GAPDH*	Forward 5’-CCACTGGTGCTGCCAAGG-3’Reverse5’-CCCTGTTGCTGTAGCCGTA-3’
H-*RRBP1*	Forward 5’-TACGACACTCAAACCTTGGGG-3’Reverse5’-GGTTGGCTAGGGCTTCTTCATA-3’
H-*ALP*	Forward 5’-GGTCATCACCAGGATTACACCA-3’Reverse5’-AAAGCCGTCAATAGCCAGGAT-3’
H-*BGLAP*	Forward 5’-CACTCCTCGCCCTATTGGC-3’Reverse5’-CCCTCCTGCTTGGACACAAAG-3’
H-*TNFSF11*	Forward 5’-CAACATATCGTTGGATCACAGCA-3’Reverse5’-GACAGACTCACTTTATGGGAACC-3’
H-*GADPH*	Forward 5’-TGCACCACCAACTGCTTAGC-3’Reverse5’-GGCATGGACTGTGGTCATGAG-3’
R-*ALP*	Forward 5’-GCTTCAGTTCCCCCTCAGTC-3’Reverse5’-TCATCAGACCCGTCGTTCAC-3’
R-*BGLAP*	Forward 5’-GAGGACCCTCTCTCTGCTCA-3’Reverse5’-GGTAGCGCCGGAGTCTATTC-3’
R-*TNFSF11*	Forward 5’-AGGCTGGGCCAAGATCTCTA-3’Reverse5’-GATAGTCCGCAGGTACGCTC-3’
R-*GADPH*	Forward 5’-GACTCTACCCACGGCAAGTT-3’Reverse5’-TGGGTTTCCCGTTGATGACC-3’

### Western blotting analysis

As described previously (22), protein extracts from MDA-MB-231, PC3, and MC3T3-E1 cells were subjected to SDS-PAGE (8-15%) and transferred to a PVDF membrane (GE Healthcare, Hertfordshire, UK), respectively. The membrane was incubated with various primary antibodies (1:1000) as required at 4°C overnight (**Table 3**), followed by the addition of the corresponding secondary antibodies at room temperature for 1 h. Enhanced chemiluminescence (ECL) detection kit was used to detect antibody reactivity (Pierce, Rockford, IL, USA).

**Table 3:** Information of antibodies.

Antibody	Manufacturer	Number
p-Smad1(Ser463/465)/5(Ser463/465)/9(Ser465/467)	CST	#13820
PERK	CST	#3192
p-PERK(Thr980)	Bioss	bs-3330R
OCN	Affinity	#DF12303
Smad1/5/9	Abcam	ab66737
BMP2	Abcam	ab14933
CHOP	Proteintech	15204-1-AP
RRBP1	Proteintech	22015-1-AP
β-actin	Sigma	A3854
Rabbit secondary antibody	Proteintech	SA00001-2
Mouse secondary antibody	Proteintech	SA0001-1

### Alizarin red S staining and evaluation of matrix mineralization

According to the manufacturer’s procedure (G1450, Solarbio, Beijing, China), MC3T3-E1 cells subjected to different treatments, including the transduction with sh*Rrbp1* and the addition of CMs from MDA-MB-231 or PC3 cells transduced with or without sh*RRBP1*, were cultured in a 1:1 mixture of double mineralized medium (α-MEM, containing 10 nM dexamethasone, 2 mM β-GP, and 50 μg/mL vitamin C) and CM from MDA-MB-231 cells or CM from PC3 cells for 28 d. The mineralized MC3T3-E1 cells were then washed with PBS three times, fixed with 4% paraformaldehyde for 15 min, washed with deionized water, dyed with 1% ARS solution for 30 min. After being washed with deionized water three times, plates were imaged under an inverted microscope (IX73, Olympus, Japan). Treated with 10% cetylpyridinium chloride destain solution for 1 h, the absorbance of the samples was measured at 562 nm using BIO-RAD 680(Hercules, CA, USA) (25).

### *In vivo* animal model of bone lesions

After being approved by the Committee on the Ethics of Animal Experiments of Xiamen University, all animal studies were conducted according to the regulations of the Institutional Animal Care and Use Committee protocol. Twenty-four 5-week-old female BALB/C nu/nu nude mice were purchased from Shanghai Slac Laboratory Animal Co.Ltd.(Shanghai, China) and randomly assigned to the two groups (12 mice each), such as MDA-MB-231 and PC3 groups. Subsequently, the MDA-MB-231 group was randomly assigned to the two sub-groups (6 mice each), such as 231-sh*Ctrl* (sh*Ctrl* and pLenti X2 Hygro/pTER shLUC (w607-1)) and 231-*shRRBP1* (sh*RRBP1* and pLenti X2 Hygro/pTER shLUC (w607-1)). Similarly, the PC3 group was randomly assigned to the two sub-groups (6 mice each), such as PC3-sh*Ctrl* (sh*Ctrl* and pLenti X2 Hygro/pTER shLUC (w607-1)) and PC3-*shRRBP1*(sh*RRBP1* and pLenti X2 Hygro/pTER shLUC (w607-1)).100 μL stable MDA-MB-231 or PC3 cells (4×10^5^/mouse) containing the control of sh*RRBP1* and pLenti X2 Hygro/pTER shLUC (w607-1) vectors or sh*RRBP1* and pLenti X2 Hygro/pTER shLUC (w607-1) vectors were injected into the tibial medullary cavity of mouse two hind legs with a 1 mL syringe, respectively. Mouse body weight was measured every 5 days. Until 55 days, mice were subjected to measurement of tumor growth with the IVIS Lumin II *in vivo* imaging (PerkinElmer, Waltham, MA, USA) by using luciferase substrates (Promega, Wisconsin, USA) (23) and then sacrificed for microcomputed tomography (micro-CT) analysis.

### Micro-CT analysis

As described previously (26–28), mouse hind legs were fixed with 4% paraformaldehyde for 48 h before micro-CT analysis. The qualitative and semi-quantitative analyses of bone destruction in the two groups were performed using high-resolution micro-CT scanning (Skyscan 1272 *In vivo* micro-CT imaging system, Bruker, Belgium). Various parameters were measured at thresholding of 85-255. Each group’s representative 3D images and mineralization mapping were carried out using SkyScan CTVox software.

### Statistical analysis

All the data are presented as the means ± SEMs. Differences between the groups were examined for statistical significance using t-test (between two groups) and one-way ANOVA following Tukey’s *post hoc* test (among three groups) with GraphPad Prism 6 software (GraphPad Software, San Diego, CA, USA). Any *p* values <0.05 were considered statistically significant. Each *in vitro* experiment was repeated three with similar results.

## Results

### RRBP1 was the sole shared high-abundance protein involving osteoblast differentiation in CMs from both MDA-MB-231 and PC3 cells

To confirm the crucial elements in the two types of cancer cells to regulate osteoblasts in the bone microenvironment, the soluble proteins in CMs from both MDA-MB-231 and PC3 cells were identified and analyzed using LC-MS/MS method. There were 530 types of proteins in the top 20% of high-abundance proteins from CM of MDA-MB-231 cells (from now CM-231), as well as 573 types of proteins from CM of PC3 cells (from now CM-PC3). 24 types of shared proteins were then identified in 530 types of proteins from CM-231 and 573 types of proteins from CM-PC3 (**Supplementary Figure 1**). The analysis results of gene ontology (GO) showed that RRBP1 was the sole protein involving osteoblast differentiation in the 24 types of shared proteins (**Tables 4**, **5**).

**Table 4:** Information of 24 types of shared proteins in CM -231 and CM-PC3.

Gene symbol	Biological process (GO)
ECH1	fatty acid beta-oxidation; fatty acid oxidation; fatty acid catabolic process
MYG1	locomotory exploration behavior; exploration behavior; locomotory behavior
MCM5	double-strand break repair *via* break-induced replication; regulation of DNA-templated DNA replication initiation; DNA unwinding involved in DNA replication
FAM118B	Cajal body organization; nuclear body organization; nucleus organization
ZC3H4	lncRNA catabolic process; regulation of lncRNA transcription; negative regulation of lncRNA transcription
RRBP1	osteoblast differentiation; ossification; translation
SUCLG2	succinyl-CoA catabolic process; succinyl-CoA metabolic process; nucleoside bisphosphate catabolic process
FBXO22	regulation of skeletal muscle fiber development; regulation of myotube differentiation; positive regulation of proteasomal ubiquitin-dependent protein catabolic process
MBNL2	regulation of alternative mRNA splicing, *via* spliceosome; regulation of mRNA splicing, *via* spliceosome; regulation of mRNA
PDXP	pyridoxal phosphate catabolic process;vitamin B6 catabolic process; actin rod assembly
OXR1	negative regulation of cellular response to oxidative stress; negative regulation of peptidyl-cysteine S-nitrosylation; cellular response to hydroperoxide
TSEN15	tRNA splicing, *via* endonucleolytic cleavage and ligation; RNA splicing, *via* endonucleolytic cleavage and ligation; tRNA processing
BICD2	minus-end-directed organelle transport along microtubule; microtubule anchoring at microtubule organizing center; microtubule anchoring
PYM1	exon-exon junction complex disassembly; nuclear-transcribed mRNA catabolic process, nonsense-mediated decay; nuclear-transcribed mRNA catabolic process
LAMTOR5	positive regulation of RNA polymerase II regulatory region sequence-specific DNA binding; regulation of RNA polymerase II regulatory region sequence-specific DNA binding; TORC1 signaling
TBC1D5	positive regulation of receptor internalization; positive regulation of receptor-mediated endocytosis; regulation of receptor internalization
PPP6R1	regulation of phosphoprotein phosphatase activity; regulation of phosphatase activity; regulation of protein dephosphorylation
POLR2F	transcription by RNA polymerase II; transcription, DNA-templated; nucleic acid-templated transcription
IGLC2	Predicted to enable antigen binding activity and immunoglobulin receptor binding activity. Predicted to be involved in several processes, including activation of immune response; defense response to other organism; and phagocytosis. Located in blood microparticle and extracellular exosome.
PPP2CA	positive regulation of microtubule binding; regulation of microtubule binding; negative regulation of tyrosine phosphorylation of STAT protein
LGALSL	like
CALML3	regulation of catalytic activity; regulation of molecular function; biological regulation
ARF5	retrograde vesicle-mediated transport, Golgi to endoplasmic reticulum; Golgi vesicle transport; intracellular protein transport
EIPR1	positive regulation of retrograde transport, endosome to Golgi; regulation of retrograde transport, endosome to Golgi; positive regulation of endocytic recycling

**Table 5:** Abundances of 24 types of shared proteins in CM -231 and CM-PC3.

Genesymbol	Coverage (%)	#Peptides	Score Sequest HT: Sequest HT	Abundances
ECH1	11	4	16.47	195.5
MYG1	33	8	40.73	185.8
MCM5	4	2	1.85	183.85
FAM118B	5	1	0	189.55
ZC3H4	1	1	18.22	187.5
RRBP1	30	32	282.8	183.45
SUCLG2	6	2	0	300
FBXO22	10	3	5.86	250.9
MBNL2	5	2	5.9	208.8
PDXP	4	1	3.07	218.95
OXR1	5	3	3.66	256.4
TSEN15	11	1	5.06	196.6
BICD2	6	4	9.55	205.55
PYM1	16	2	5.76	230.7
LAMTOR5	37	2	3.53	214.25
TBC1D5	2	1	2.52	210.25
PPP6R1	5	2	6.11	210.4
POLR2F	6	1	2.25	300
IGLC2	32	2	59.19	300
PPP2CA	44	9	159.43	300
LGALSL	6	1	0	300
CALML3	19	2	67.02	274.25
ARF5	42	5	55.62	300
EIPR1	3	1	6.11	300

The underline was used to highlight "RRBP1".

### CMs from both MDA-MB-231 and PC3 cells transduced with sh*RRBP1* enhanced the osteoblastic phenotype expression in MC3T3-E1 cells

**Figure 1:**
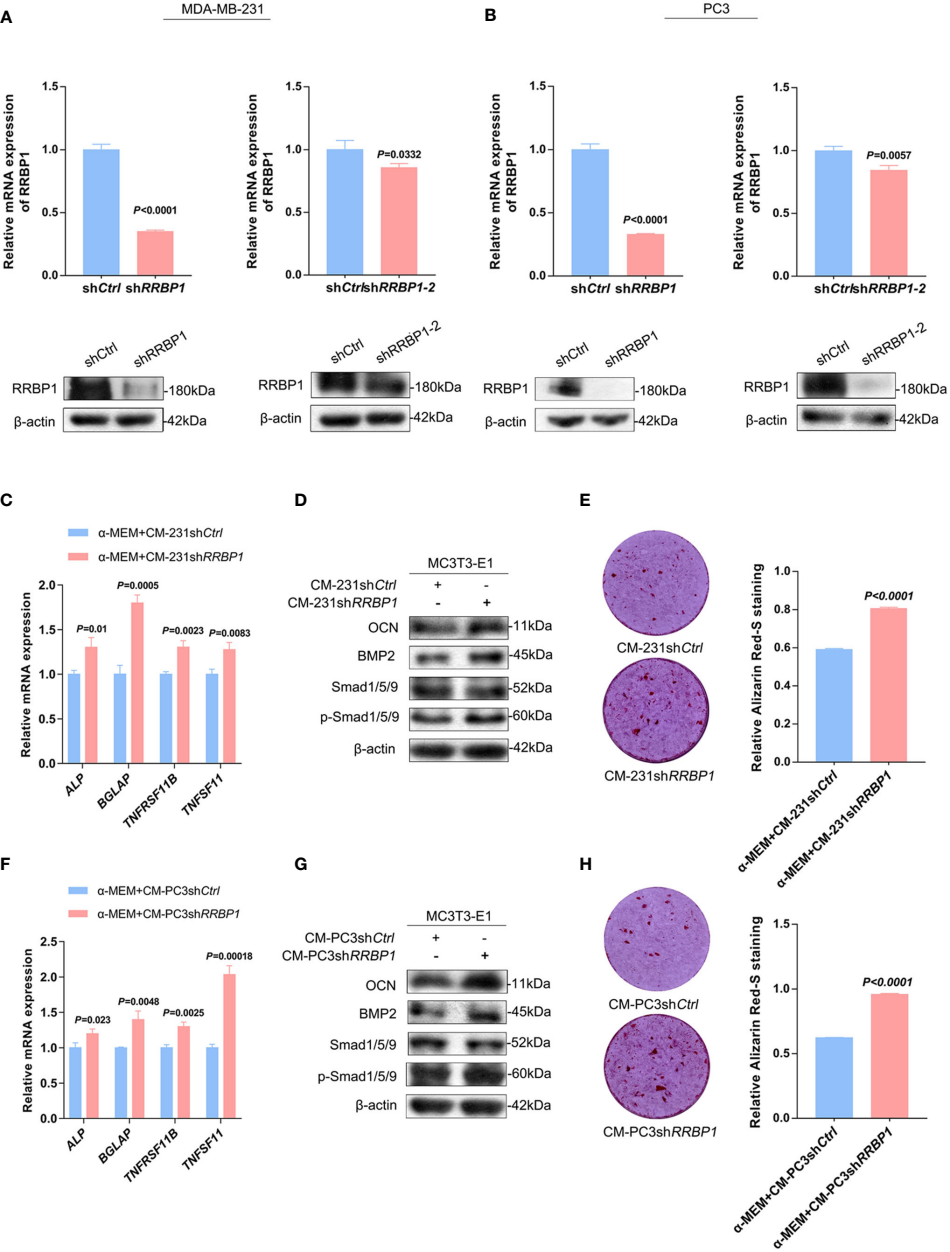
The effect of CM from MDA-MB-231 or PC3 cells transduced with sh*RRBP1* on the osteoblastic phenotype expression in MC3T3-E1 cells. **(A)** MDA-MB-231 cells were transduced with sh*RRBP1* or sh*RRBP1-*2 vectors, respectively. The relative mRNA and protein levels of RRBP1 were measured *via* qPCR and western blotting assays, respectively. **(B)** PC3 cells were transduced with sh*RRBP1* or sh*RRBP1-*2 vectors, respectively. The relative mRNA and protein levels of RRBP1 were measured *via* qPCR and western blotting assays, respectively. **(C, D)** MC3T3-E1 cells were cultured in α-MEM+CM-231sh*Ctrl* and α-MEM+CM-231sh*RRBP1* for 7 d, respectively. Relative mRNA levels of *ALP, BGLAP, TNFRSF11B*, and *TNFSF11* were measured *via* qPCR assay **(C)**. The protein levels of OCN, BMP2, Smad1/5/9, p-Smad1/5/9, and β-actin were measured *via* western blotting analysis **(D)**. **(E)** MC3T3-E1 cells were cultured in α-MEM+CM-231 sh*Ctrl* and α-MEM+CM-231sh*RRBP1* for 28 d, respectively, followed by ARS staining and evaluation of mineralization. **(F, G)** MC3T3-E1 cells were cultured in α-MEM+CM-PC3sh*Ctrl* and α-MEM+CM-PC3sh*RRBP1* for 7 d, respectively. Relative mRNA levels of *ALP, BGLAP, TNFRSF11B*, and *TNFSF11 were* measured *via* qPCR assay **(F)**. The protein levels of OCN, BMP2, Smad1/5/9, p-Smad1/5/9, and β-actin were measured *via* western blotting analysis **(G)**. **(H)** MC3T3-E1 cells were cultured in α-MEM+CM-PC3sh*Ctrl* and α-MEM+CM-PC3sh*RRBP1* for 28 d, respectively, followed by ARS staining and evaluation of mineralization. The data are representative of three independent experiments.

The expression levels of RRBP1 in breast and prostatic adenocarcinoma tumor tissues were analyzed *via* Gene Expression Profiling Interactive Analysis (GEPIA) database. The results showed a significantly higher expression of RRBP1 in tumor tissues(T), compared with normal tissues(N) (**Supplementary Figure 2A**). Meanwhile, we observed that the protein expression levels of RRBP1 in the two-type cancer cells were higher than that in MC3T3-E1 cells (**Supplementary Figure 2B**). Thus, MDA-MB-231 and PC3 cells were then simultaneously transduced with the short hairpin RNA targeting the *RRBP1* vector (sh*RRBP1*) for subsequent experiments. As shown in **Figures 1A, B**, the mRNA and protein levels of RRBP1 were downregulated in MDA-MB-231 or PC3 cells transduced with sh*RRBP1* or sh*RRBP1*-2, compared with the control vector (sh*Ctrl*). MC3T3-E1 cells were then cultured with a mixed medium (1:1 mixture of double mineralized medium(α-MEM) and CMs from two-type cancer cells that were transduced with sh*RRBP1*). The mRNA levels of *ALP* (alkaline phosphatase, ALP, gene name ALP, an early differentiation marker*), BGLAP* (osteocalcin, OCN, gene name BGLAP, a later differentiation marker), *TNFRSF11B* (osteoprotegerin, OPG, gene name *TNFRSF11B*, a soluble decoy receptor for *TNFSF11*), and *TNFSF11*(receptor activator of nuclear factor-κB ligand, RANKL, gene name TNFSF11) were measured using qPCR assay. As shown in **Figure 1C**, CM from MDA-MB-231 cells transduced with sh*RRBP1* (CM-231sh*RRBP1*) resulted in significantly increased *ALP, BGLAP, TNFRSF11B*, and *TNFSF11* mRNA levels in MC3T3-E1 cells, compared with CM-231sh*Ctrl*. Meanwhile, the protein expression levels of some signal molecules related to osteoblastic phenotype, such as OCN, bone morphogenetic protein 2 (BMP2), and small mother against decapentaplegic (SMAD) 1/5/9 (Smad1/5/9), and matrix mineralization were assessed using western blotting analysis and ARS staining assay, respectively. Compared with CM-231sh*Ctrl*, CM-231sh*RRBP1* increased the levels of OCN, BMP2, and p-Smad1/5/9 and matrix mineralization (**Figures 1D, E**). Similarly, CM from PC3 cells transduced with sh*RRBP1* (CM-PC3sh*RRBP1*) resulted in significantly increased *ALP*, *BGLAP*, *TNFRSF11B*, and *TNFSF11* mRNA levels in MC3T3-E1 cells, compared with CM-PC3sh*Ctrl* (**Figure 1F**), as well as the levels of OCN, BMP2, p-Smad1/5/9 (**Figure 1G**) and mineralization (**Figure 1H**).

### Injection with both MDA-MB-231 and PC3 cells transduced with sh*RRBP1* ameliorated the bone lesions in nude mice

**Figure 2:**
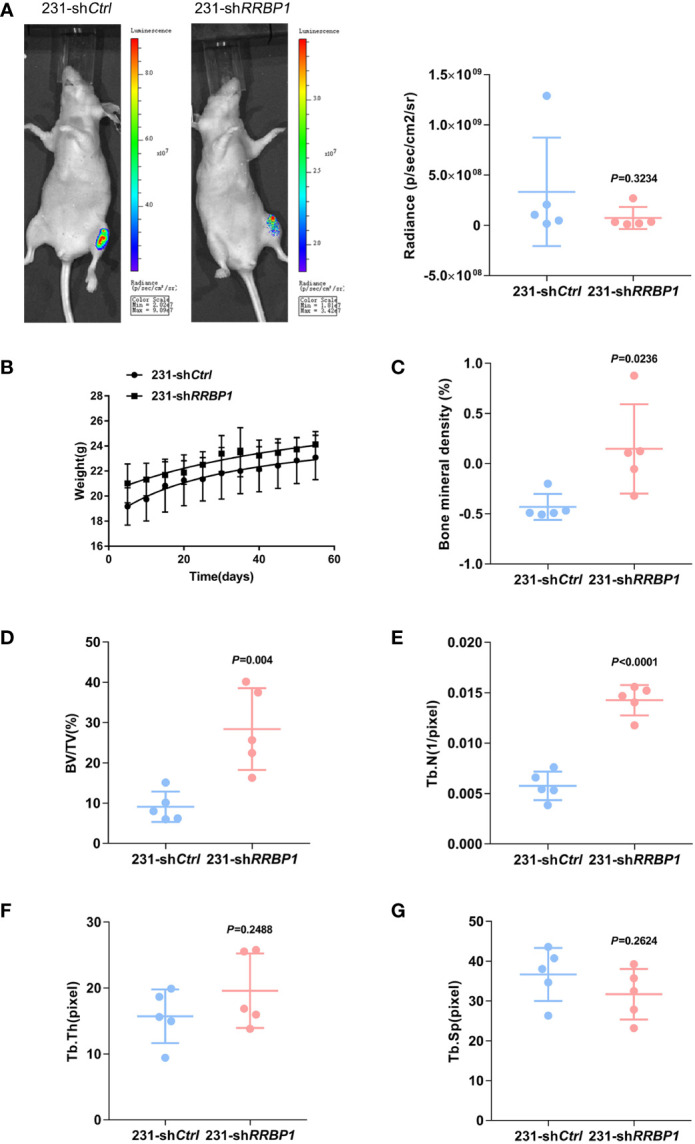
The effect of injection with MDA-MB-231 transduced with shRRBP1 into the proximal metaphysic of the tibia on bone mass in a nude mouse model. MDA-MB-231 cells transduced with sh*RRBP1* were injected into the proximal metaphysic of the tibia in nude mice for 8 w. **(A)** The radiance (p/sec/cm2/sr) of tumor mass was measured using the IVIS Lumin II *in vivo* imagining. **(B)** Mouse body weight was measured and analyzed using GraphPad Prism 6 software. **(C–G)** Quantitative analysis of structural parameters of the tibia by high-resolution micro-CT scanning: bone mineral density **(C)**, bone volume/total tissue volume (BV/TV) **(D)**, trabecular number (Tb. N) **(E)**, trabecular thickness (Tb. Th) **(H)**, and Trabecular Space (Tb. Sp) **(G)**.

**Figure 3:**
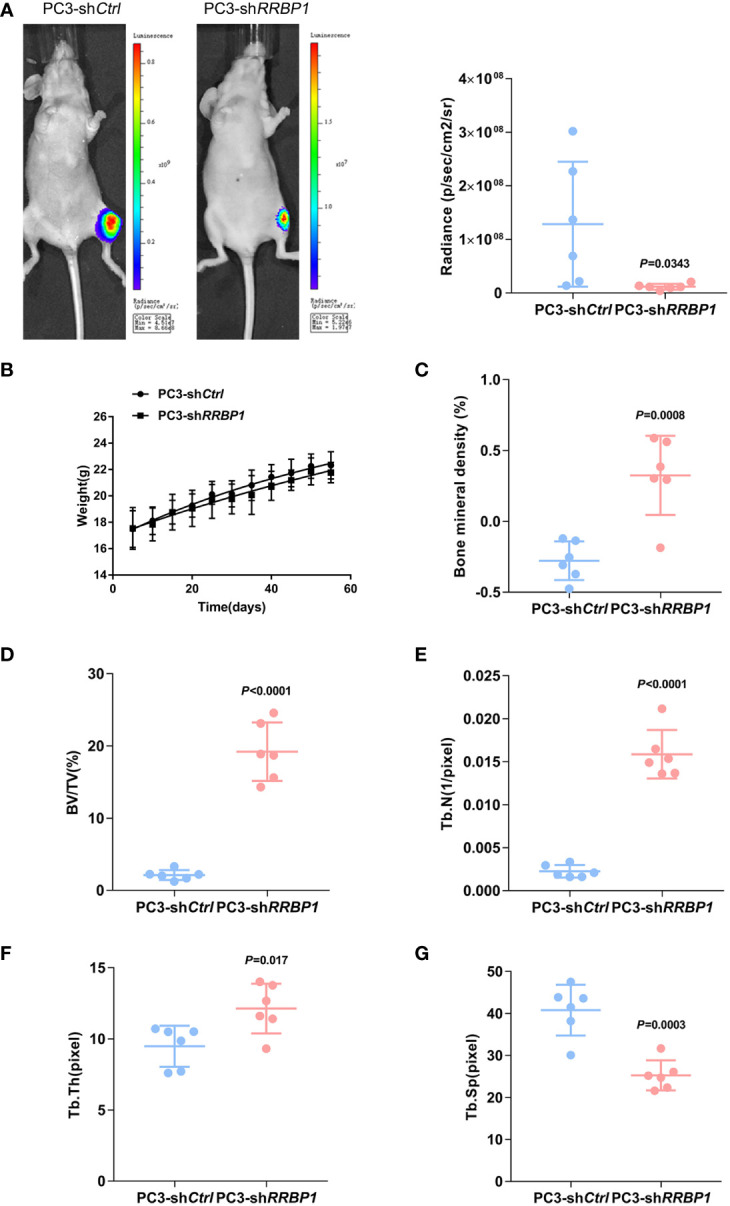
The effect of injection with PC3 transduced with shRRBP1 into the proximal metaphysic of the tibia on bone mass in a nude mouse model. PC3 cells transduced with sh*RRBP1* were injected into the proximal metaphysic of the tibia in nude mice for 8 w. **(A)** The radiance (p/sec/cm2/sr) of tumor mass was measured using the IVIS Lumin II *in vivo* imagining. **(B)** Mouse body weight was measured and analyzed using GraphPad Prism 6 software. **(C–G)** Quantitative analysis of structural parameters of the tibia by high-resolution micro-CT scanning: bone mineral density **(C)**, bone volume/total tissue volume (BV/TV) **(D)**, trabecular number (Tb. N) **(E)**, trabecular thickness (Tb. Th) **(H)**, and Trabecular Space (Tb. Sp) **(G)**.

MDA-MB-231 and PC3 cells transduced with sh*RRBP1* were injected into the proximal tibial metaphysic in nude mice for 8 w, respectively (**Supplementary Figure 3A**). As shown in **Figures 2A, B**, there was no significant difference in tumor size and mouse body weight between the 231-sh*Ctrl* and 231-sh*RRBP1* groups. Micro-CT images were shown in **Supplementary Figures 3B, C**. The quantitative results of micro-CT analysis showed significantly increased bone mineral density, bone volume fraction (BV/TV), and trabecular number (Tb. N), and no significant alterations in trabecular thickness (Tb. Th) and trabecular space (Tb. Sp) in the 231-sh*RRBP1* group, compared with the 231-sh*Ctrl* group (**Figures 2C–G**). In the nude mice injected with PC3 cells transduced with sh*RRBP1* for 8 w, there was a slightly decreased tumor size in the PC3-sh*RRBP1* group, compared with the PC3-sh*Ctrl* group (**Figure 3A**, *p*=0.0343), although there was no difference in mouse body weight (**Figure 3B**). Quantitatively, bone mineral density, BV/TV, Tb. N, and Tb. Th in the PC3-sh*RRBP1* group were significantly higher than that in the PC3-sh*Ctrl* group, while Tb. Sp in the PC3-sh*RRBP1* group was significantly lower than that in the PC3-sh*Ctrl* group (**Figures 3C–G**).

### Enhancement of ER stress contributed to the regulation of sh*RRBP1* from CM-231 or CM-PC3 on the osteoblastic phenotype expression in MC3T3-E1 cells

**Figure 4:**
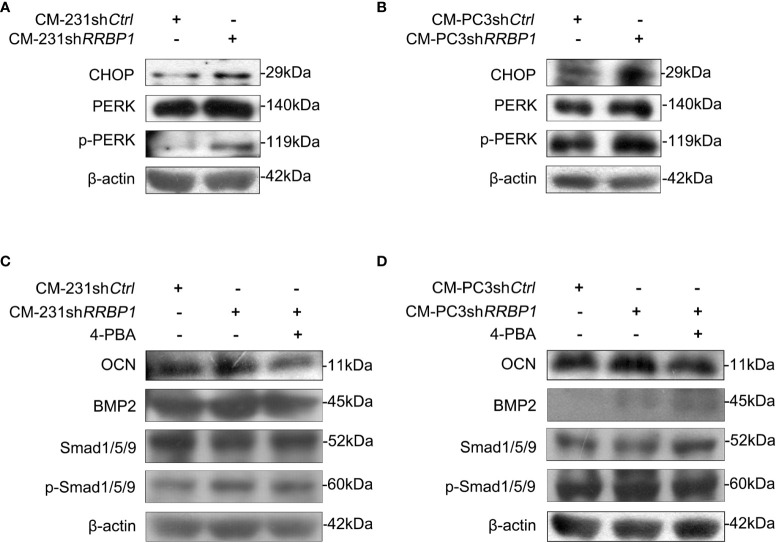
The involvement of ER stress in increased expression of osteoblastic phenotype mediated by CM from MDA-MB-231 or PC3 cells transduced with sh*RRBP1*. **(A, B)** MC3T3-E1 cells were cultured in α-MEM+CM-231sh*Ctrl* and α-MEM+CM-231sh*RRBP1* or α-MEM+CM-PC3sh*Ctrl* and α-MEM+CM-PC3sh*RRBP1* for 7 d, respectively. The protein levels of CHOP, PERK, p-PERK, and β-actin were measured *via* western blotting analysis. **(C, D)** MC3T3-E1 cells were cultured in α-MEM+CM-231sh*Ctrl* and α-MEM+CM-231sh*RRBP1* or α-MEM+CM-PC3sh*Ctrl* and α-MEM+CM-PC3sh*RRBP1* for 5 d, followed by the treatment of 4-PBA (0.5 mM) for 2 d. The protein levels of OCN, BMP2, Smad1/5/9, p-Smad1/5/9, and β-actin were measured *via* western blotting analysis. The data are representative of three independent experiments.

As a ribosome binding protein, RRBP1 is a member of the ER stress response (16), some ER stress biomarkers, such as the transcription factor C/EBP homologous protein (CHOP) and the double-stranded RNA-dependent protein kinase (PKR)-like ER kinase (PERK), were then assessed in MC3T3-E1 cells cultured by CMs from sh*RRBP1*-transduced two-type cancer cells. The results in **Figure 4A** showed that CMs from MDA-MB-231 cells transduced with sh*RRBP1*(CM-231sh*RRBP1*) led to increased expression levels of CHOP, p-PERK, and PERK in MC3T3-E1 cells, compared with CM-231sh*Ctrl*. Similar results were observed in MC3T3-E1 cells cultured by CMs from PC3 cells transduced with sh*RRBP1* (CM-PC3sh*RRBP1*) (**Figure 4B**). To confirm the role of ER stress in regulating osteoblastic phenotype, ER stress inhibitor 4-phenylbutiric acid (4-PBA) was added in the mixed medium prior to MC3T3-E1 cells culture. As shown in **Figure 4C**, the increased expression levels of OCN, BMP2, and p-Smad1/5/9 induced by CM-231sh*RRBP1* were down-regulated by the addition of 4-PBA in MC3T3-E1 cells. Similarly, CM-PC3sh*RRBP1* also increased the levels of OCN, BMP2, and p-Smad1/5/9 and the addition of 4-PBA partially reduced the increased levels of OCN and p-Smad1/5/9, except the level of BMP2 that was up-regulated by 4-PBA (**Figure 4D**).

### Effect of RRBP1 depletion with sh*Rrbp1* on the osteoblastic phenotype expression and matrix mineralization in MC3T3-E1 cells were partially offset by CMs from both MDA-MB-231 and PC3 cells

**Figure 5:**
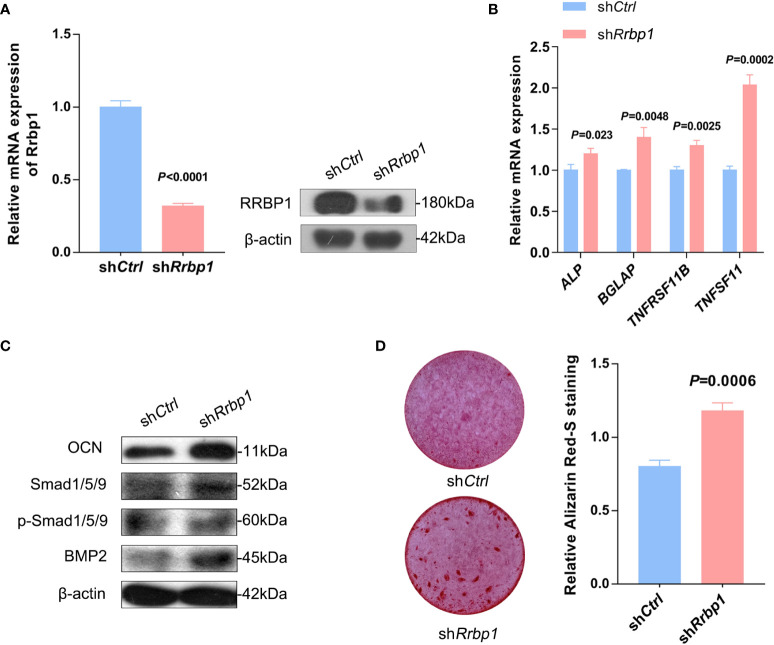
The osteoblastic phenotype expression in MC3T3-E1 cells transduced with sh*Rrbp1*. **(A–C)** MC3T3-E1 cells transduced with sh*Rrbp1* were cultured with a mineralized medium for 7 d. The relative mRNA level of *Rrbp1* was measured *via* qPCR assay and the protein levels of RRBP1 and β-actin were measured *via* western blotting analysis **(A)**. Relative mRNA levels of *ALP, BGLAP, TNFRSF11B*, and *TNFSF11* were measured *via* qPCR assay **(B)**. The protein levels of OCN, BMP2, Smad1/5/9, p-Smad1/5/9, and β-actin were measured *via* western blotting analysis **(C)**. **(D)** MC3T3-E1 cells transduced with sh*Rrbp1* were cultured with a mineralized medium for 28 d, followed by ARS staining. Left panel: representative images; right panel: absorbance values at 575 nm of different groups. The data are representative of three independent experiments.

**Figure 6:**
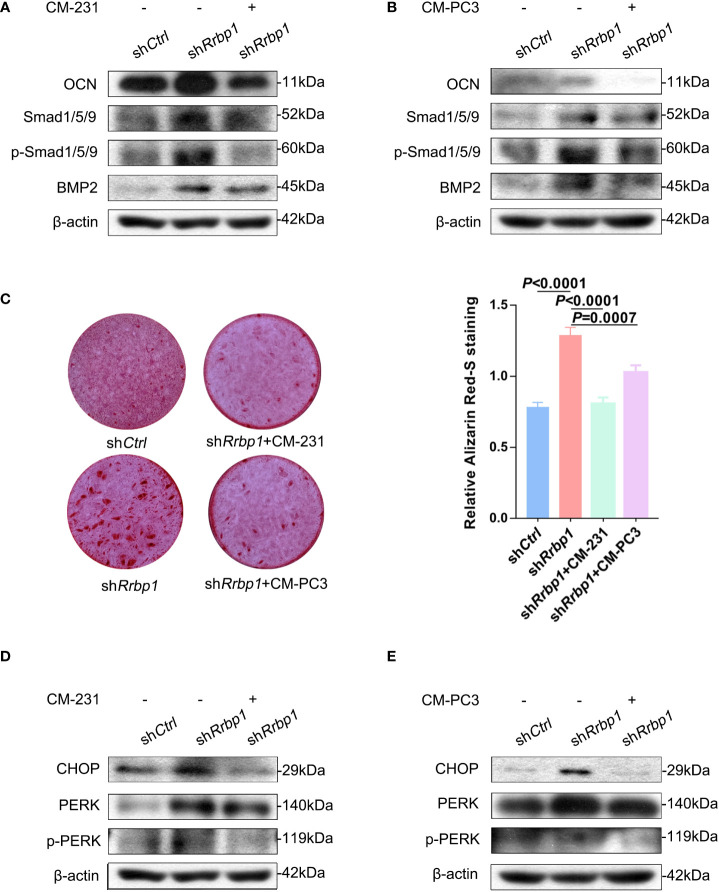
The effect of CM from MDA-MB-231 or PC3 cells on the osteoblastic phenotypic expression in MC3T3-E1 cells transduced with sh*Rrbp1*. **(A, B)** MC3T3-E1 cells transduced with sh*Rrbp1* were cultured in α-MEM+CM-231 or α-MEM+CM-PC3 for 7d, respectively. The protein levels of OCN, BMP2, Smad1/5/9, p-Smad1/5/9, and β-actin were measured *via* western blotting analysis. **(C)** MC3T3-E1 cells transduced with sh*Rrbp1* were cultured in α-MEM+ CM-PC3 and α-MEM+CM-PC3 for 28 d, respectively, followed by ARS staining. Left panel: representative images; right panel: absorbance values at 575 nm of different groups. **(D, E)** MC3T3-E1 cells transduced with sh*Rrbp1* were cultured in α-MEM+CM-231 or α-MEM+CM-PC3 for 7d. The protein levels of CHOP, IREα, p-IREα, PERK, p-PERK, and β-actin were measured *via* western blotting analysis. The data are representative of three independent experiments.

As shown in **Figure 5A**, RRBP1 depletion with sh*Rrbp1*(sh*Rrbp1*) suppressed the mRNA and protein expression of RRBP1 in MC3T3-E1 cells. The transduction of sh*Rrbp1* resulted in significantly increased *ALP*, *BGLAP*, *TNFRSF11B*, and *TNFSF11* mRNA levels, compared with the sh*Ctrl* group (**Figure 5B**), accompanied with increased levels of OCN, Smad1/5/9, p-Smad1/5/9, and BMP2 (**Figure 5C**) and matrix mineralization (**Figure 5D**). To further confirm whether RRBP1 from CM-231 and CM-PC3 affected the osteoblastic phenotype, CM-231 or CM-PC3, as a biological reagent, was added in the medium for culturing MC3T3-E1 cells transduced with sh*Rrbp1*. It was observed that compared with the sh*Rrbp1* group, the addition of CM-231 reduced the increased levels of OCN, Smad1/5/9, p-Smad1/5/9, and BMP2 and matrix mineralization induced by sh*Rrbp1* in MC3T3-E1 cells (**Figures 6A, C**), as well as CM-PC3 (**Figures 6B, C**). The increased levels of CHOP, PERK, and p-PERK caused by sh*Rrbp1* were attenuated by the addition of either CM-231 or CM-PC3, compared with the sh*Rrbp1* group (**Figures 6D, E**).

## Discussion

Despite it is substantially expressed in bone metastatic cancer cells, the role of RRBP1 in regulating the bone microenvironment was obscure. In this study, our findings showed that RRBP1 was the sole type of shared protein involving osteoblast differentiation in the top 20% of high-abundance proteins from CM-231 and CM-PC3. CMs from both MDA-MB-231 and PC3 cells transduced with sh*RRBP1* significantly increased the mRNA levels of *ALP*, *BGLAP*, *TNFRSF11B*, and *TNFSF11* in MC3T3-E1 cells, as well as matrix mineralization and OCN, BMP2, and p-Smad1/5/9 protein levels. These results indicated that either MDA-MB-231 cell- or PC3 cell-derived RRBP1 suppressed the osteoblastic phenotype expression, at least partially, by interrupting the BMP2/Smad1/5/9 pathway, which is a conserved signaling pathway and plays a key role in osteoblast activation and extracellular matrix calcification (6, 29). Furthermore, the increased mineralization and OCN, p-Smad1/5/9, and BMP2 levels in MC3T3-E1 cells caused by sh*Rrbp1* transduction were offset partially by CMs from MDA-MB-231 and PC3 cells. Additionally, we observed that the injection of both MDA-MB-231 and PC3 cells transduced with sh*RRBP1* into the proximal metaphysis of the tibia partially significantly increased bone mineral density, BV/TV, and Tb. N in nude mice. As for the inconsistent Tb. Th and Tb. Sp in the tibia of nude mice between the injection of two-type cancer cells transduced with *shRRBP1*, it might be associated with the differential regulation of other cancer-secreted extracellular factors in the bone environments. Taken together, it is demonstrated that as the sole shared high-abundance protein involving osteoblast differentiation from CMs of MDA-MB-231 and PC3 cells, RRBP1 depletion with sh*RRBP1* in cancer cells enhanced the osteoblastic phenotype expression and ameliorated the bone lesions induced by bone metastatic cancer cells. Therefore, our findings provided novel key information regarding the role of RRBP1 in bone metastatic cancer. RRBP1 of bone metastatic cancer cells might exacerbate bone lesions by suppressing the osteoblastic phenotype, suggesting that RRBP1 represents an attractive target for the treatment of the bone lesions induced by bone metastatic cancers. Considering the role of osteoclasts in the bone lesions, the other shared high-abundance proteins from CM-231 and CM-PC3 involving osteoclast function might be worthy of attention in our future studies. Combined with recent studies on drug delivery systems (30–32), targeting these high-abundance proteins in bone metastatic cancer cells would be a potential approach for ameliorating bone metastatic lesions.

ER stress induced by various physiological and pathological stimuli could lead to the accumulation of the unfolded or misfold response proteins in the ER lumen, thereby representing a fundamental threat to cell viability (33, 34). In response to ER stress, an adaptive signaling pathway in the ER is triggered to copy with the stress through attenuating protein synthesis, clearing the unfolded/misfolded proteins, and increasing the capacity of ER to fold protein (35). This process is referred to as the unfolded protein response (UPR), which is controlled by three major sensors: IRE1(inositol-requiring 1), ATF6(activating transcription factor 6), and PERK (double-strand RNA-dependent protein kinase (PKR)-like ER kinase). These three ER stress sensors are normally bound by the ER chaperone GRP78/BIP (35–37). When accumulating misfolded proteins in the ER lumen engage GRP78, the three sensors are released to activate their downstream pathways, including PERK/CHOP, thereby relieving ER stress and restoring ER function (35–37). It has been determined that ER stress influences osteoblast differentiation. On the hand, the inducement of ER stress impairs osteoblast differentiation (38, 39). For example, the deficiency of ADP-ribosylation-like factor 6 interacting protein 5 (Arl6ip5) impairs osteoblast differentiation *via* the inducement of ER stress and enhancement of ER stress-mediated apoptosis (38). Defective autophagy in osteoblasts induces ER stress and causes remarkable bone loss (39). On the other hand, the inducement of ER stress facilities osteoblastic differentiation (34, 40). The treatment of cranial immature osteoblasts with BMP2 induces mild ER stress involving the facilitation of osteogenesis, associated with a high demand for the synthesis and secretion of bone matrix proteins (34). Dr. Chen and his colleagues address that ER stress occurred after tooth extraction and regulating the degree of ER stress can promote bone healing in tooth extraction sockets by increasing the expression of Runx2 and ALP (40). Hence, the indefinite influence of ER stress on osteoblast metabolism may be due to a difference in its degree. Our findings showed that RRBP1 depletion increased CHOP and p-PERK levels in MC3T3-E1 cells cultured with CM from MDA-MB-231 and PC3 cells transduced with shRRBP1 vector, as well as in MC3T3-E1 cells transduced with sh*Rrbp1*. These results indicated that RRBP1 depletion induced ER stress *via* increasing p-PERK and CHOP in MC3T3-E1 cells, similar to a previous study that RRBP1 knockdown by shRNAs causes ER stress in lung cancer cells (16). Furthermore, it was observed that the addition of ER stress inhibitor 4-PBA partially attenuated the promoting effect of CMs from sh*RRBP1*-transduced two-type cancer cells on OCN and p-Smad1/5/9 levels in MC3T3-E1 cells, implicating that RRBP1 depletion-induced ER stress partially aided in the enhancement of osteoblastic phenotype expression in MC3T3-E1 cell. Therefore, it is suggested that RRBP1 depletion might induce mild ER stress, thereby enhancing the osteoblastic phenotype, consistent with the previous studies that mild ER stress facilities osteoblastic differentiation (34, 40). Additionally, increased CHOP, PERK, OCN, p-Smad1/5/9, and BMP2 levels and matrix mineralization induced by sh*Rrbp1* in MC3T3-E1 cells were offset partially by CMs from both MDA-MB-231 and PC3 cells, providing strong evidence for the involvement of RRBP1 from bone metastatic cells in suppressing the osteoblastic phenotype expression associated with decreased ER stress.

## Conclusion

In summary, RRBP1 was the sole shared high-abundance protein involving osteoblast differentiation in CMs from both MDA-MB-231 and PC3 cells. CMs from both MDA-MB-231 and PC3 cells transduced with sh*RRBP1* enhanced the osteoblastic phenotype expression in MC3T3-E1 cells. Meanwhile, the injection of MDA-MB-231 and PC3 cells transduced with sh*RRBP1* into the tibia medullary cavity partially ameliorated the bone lesions in the tibia of nude mice. Furthermore, RRBP1 depletion-induced ER stress aided in the enhancement of osteoblastic phenotype expression, exhibiting the increased p-PERK and CHOP levels. Therefore, depletion of RRBP1 with shRRBP1 in bone metastatic cancer cells could boost the osteoblastic phenotype expression and ameliorate the bone lesions, partially *via* the enhancement of ER stress. Bone metastatic cancer-secreted RRBP1 may play an important role in regulating the bone microenvironment, *via* targeting osteoblasts.

## Data availability statement

The original contributions presented in the study are included in the article/**Supplementary Material**. Further inquiries can be directed to the corresponding authors.

## Ethics statement

The animal study was reviewed and approved by the Committee on the Ethics of Animal Experiments of Xiamen University.

## Author contributions

RC: Data curation, formal analysis, experiment, methodology, writing-original draft of materials and methods. YW: Data curation, software, experiment, methodology. YX: Data curation, experiment. YH: Software, methodology. QL: Resources, funding acquisition. CX: Conceptualization, funding acquisition, writing-original draft of discussion section. BZ: Conceptualization, resources, supervision, writing-original draft of the other sections, writing-review & editing. All authors contributed to the article and approved the submitted version.

## Supplementary material

Supplementary Figure 1 Identification of RRBP1 in CM from MDA-MB-231 or PC3 cells using LC-MS/MS analysis. The soluble mediators in CMs from MDA-MB-231 (CM-231) or PC3(CM-PC3) cells were identified and analyzed using LC-MS/MS analysis, respectively. 24 types of shared proteins were then identified in 530 types of proteins from CM-231 and 573 types of proteins from CM-PC3.

Supplementary Figure 2 The expression of RRBP1 in related tissues and cells. **(A)** The expression levels of RRBP1 in breast and prostate tumor tissues were analyzed *via* Gene Expression Profiling Interactive Analysis (GEPIA) database. **(B)** The protein expression levels of RRBP1 in MDA-MB-231, PC3, and MC3T3-E1 cells were detected *via* western blotting analysis.

Supplementary Figure 3 The effect of injection with MDA-MB-231 or PC3 cells transduced with sh*RRBP1* into the proximal metaphysic of the tibia on the bone lesions in a nude mice model. **(A)** Schematic diagram illustrating the injection site of MDA-MB-231 cells and PC3 cells transduced with sh*RRBP1* in a nude mice model. **(B, C)** Representative micro-CT images of the tibia showing bone mass using high-resolution micro-CT scanning. The effect of injection with MDA-MB-231 cells transduced with sh*RRBP1* into the proximal metaphysic of the tibia on the bone lesions in a nude mice model **(B)**. The effect of injection with PC3 cells transduced with sh*RRBP1* into the proximal metaphysic of the tibia on the bone lesions in a nude mice model **(C)**.
