# Supplementary figures and images for: RRBP1 depletion of bone metastatic cancer cells contributes to enhanced expression of the osteoblastic phenotype
Source: Front Oncol. 2022 Dec 9;12:1005152. doi: 10.3389/fonc.2022.1005152 (PMC9782440; doi:10.3389/fonc.2022.1005152)

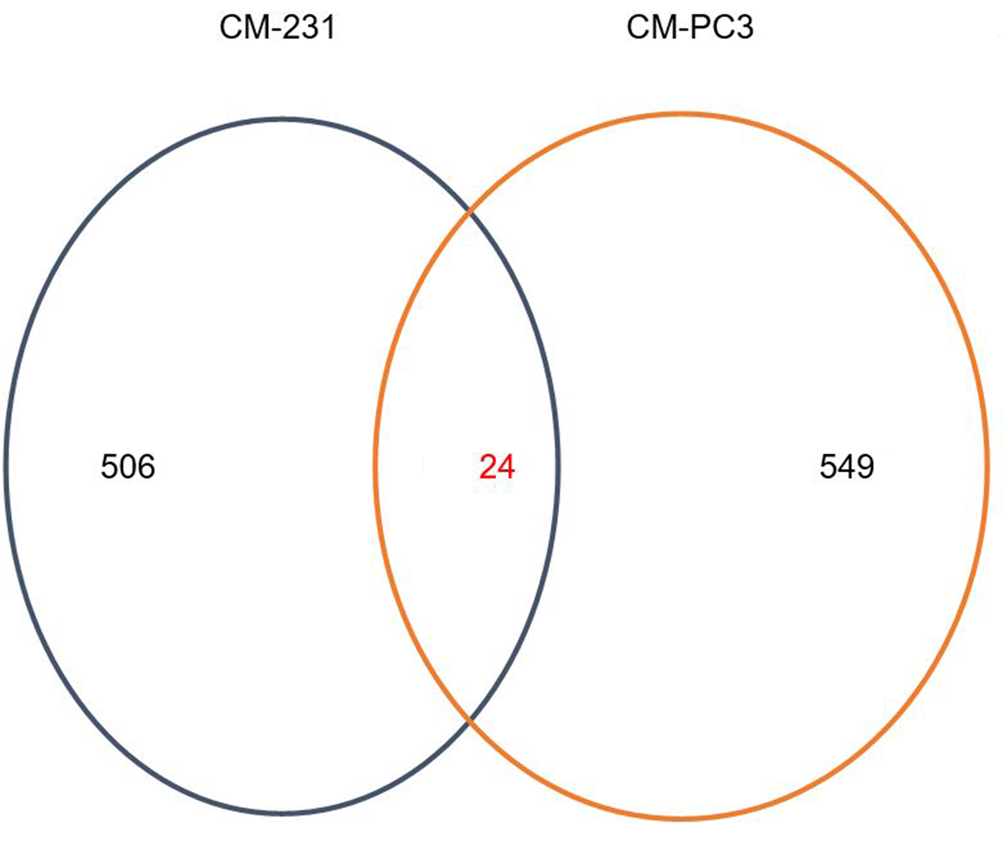

Supplement: Supplementary Figure 1 — Identification of RRBP1 in CM from MDA-MB-231 or PC3 cells using LC-MS/MS analysis. The soluble mediators in CMs from MDA-MB-231 (CM-231) or PC3(CM-PC3) cells were identified and analyzed using LC-MS/MS analysis, respectively. 24 types of shared proteins were then identified in 530 types of proteins from CM-231 and 573 types of proteins from CM-PC3. [file Image_1.tiff]

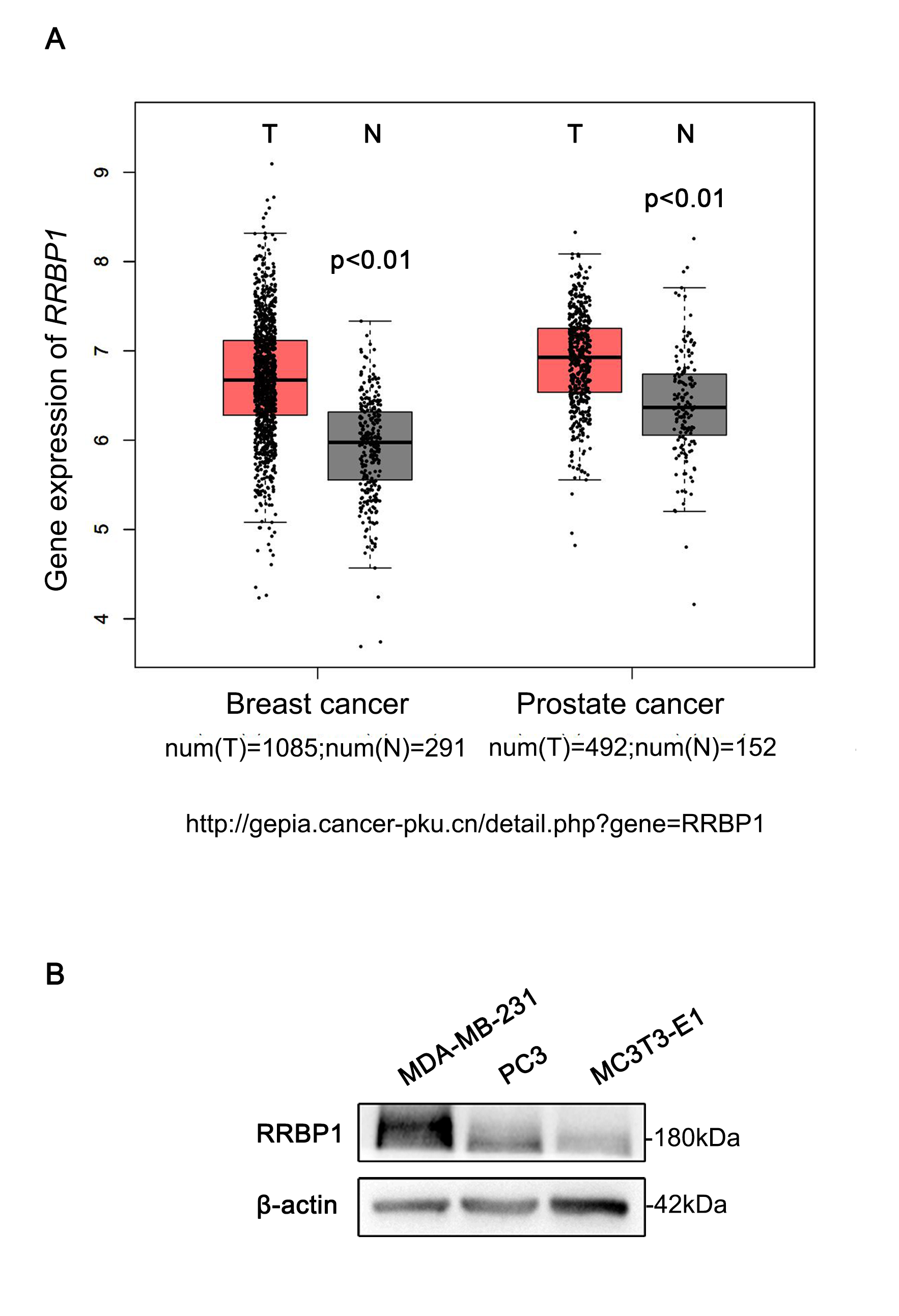

Supplement: Supplementary Figure 2 — The expression of RRBP1 in related tissues and cells. (A) The expression levels of RRBP1 in breast and prostate tumor tissues were analyzed via Gene Expression Profiling Interactive Analysis (GEPIA) database. (B) The protein expression levels of RRBP1 in MDA-MB-231, PC3, and MC3T3-E1 cells were detected via western blotting analysis. [file Image_2.tiff]

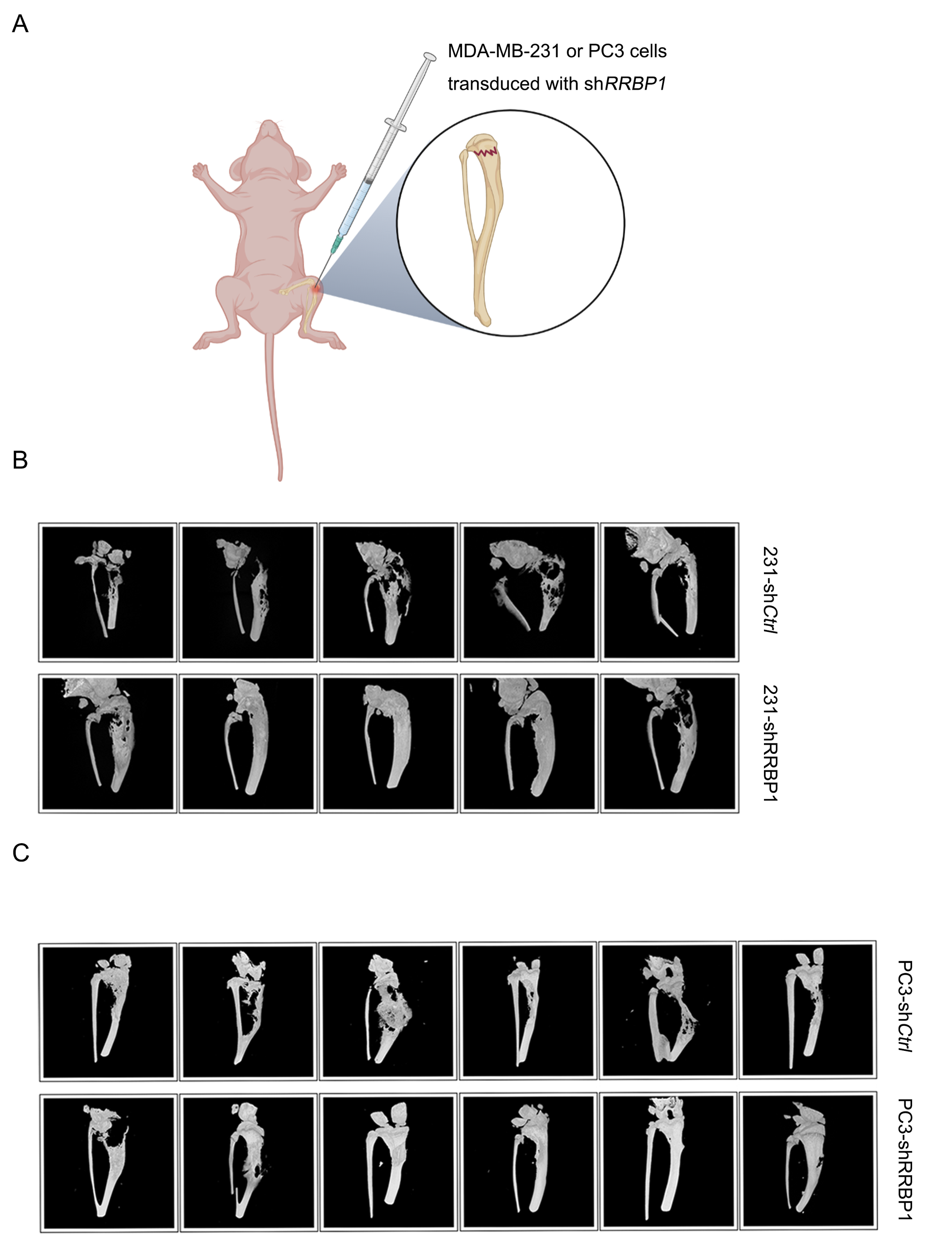

Supplement: Supplementary Figure 3 — The effect of injection with MDA-MB-231 or PC3 cells transduced with shRRBP1 into the proximal metaphysic of the tibia on the bone lesions in a nude mice model. (A) Schematic diagram illustrating the injection site of MDA-MB-231 cells and PC3 cells transduced with shRRBP1 in a nude mice model. (B, C) Representative micro-CT images of the tibia showing bone mass using high-resolution micro-CT scanning. The effect of injection with MDA-MB-231 cells transduced with shRRBP1 into the proximal metaphysic of the tibia on the bone lesions in a nude mice model (B). The effect of injection with PC3 cells transduced with shRRBP1 into the proximal metaphysic of the tibia on the bone lesions in a nude mice model (C). [file Image_3.tiff]
